# The effectiveness and feasibility of an online educational program for improving evidence-based practice literacy: an exploratory randomized study of US chiropractors

**DOI:** 10.1186/s12998-016-0109-8

**Published:** 2016-08-02

**Authors:** Michael Schneider, Roni Evans, Mitchell Haas, Matthew Leach, Louise Delagran, Cheryl Hawk, Cynthia Long, Gregory D. Cramer, Oakland Walters, Corrie Vihstadt, Lauren Terhorst

**Affiliations:** 1 School of Health and Rehabilitation Sciences, Department of Physical Therapy, Clinical and Translational Science Institute, University of Pittsburgh, Pittsburgh, PA USA; 2 Center for Spirituality and Healing, Integrative Health and Wellbeing Research Program, University of Minnesota, Minneapolis, MN USA; 3 University of Western States, Portland, OR USA; 4 School of Nursing and Midwifery, University of South Australia, Adelaide, SA Australia; 5 Center for Spirituality and Healing, University of Minnesota, Minneapolis, MN USA; 6 Chair, Scientific Commission, Council on Chiropractic Guidelines and Practice Parameters, Lexington, SC USA; 7 Palmer Center for Chiropractic Research, Palmer College of Chiropractic, Davenport, IA USA; 8 National University of Health Sciences, Lombard, IL USA; 9 The Commonwealth Medical College, Scranton, PA USA; 10 School of Health and Rehabilitation Sciences, Department of Occupational Therapy, Clinical and Translational Science Institute, University of Pittsburgh, Pittsburgh, PA USA

**Keywords:** Evidence-based practice, Chiropractic, Online education, Knowledge translation

## Abstract

**Background:**

Online education programs are becoming a popular means to disseminate knowledge about evidence-based practice (EBP) among healthcare practitioners. This mode of delivery also offers a viable and potentially sustainable solution for teaching consistent EBP content to learners over time and across multiple geographical locations. We conducted a study with 3 main aims: 1) develop an online distance-learning program about the principles of evidence-based practice (EBP) for chiropractic providers; 2) test the effectiveness of the online program on the attitudes, skills, and use of EBP in a sample of chiropractors; and 3) determine the feasibility of expanding the program for broader-scale implementation. This study was conducted from January 2013 to September 2014.

**Methods:**

This was an exploratory randomized trial in which 293 chiropractors were allocated to either an online EBP education intervention or a waitlist control. The online EBP program consisted of 3 courses and 4 booster lessons, and was developed using educational resources created in previous EBP educational programs at 4 chiropractic institutions. Participants were surveyed using a validated EBP instrument (EBASE) with 3 rescaled (0 to 100) subscores: Attitudes, Skills, and Use of EBP. Multiple regression was used to compare groups, adjusting for personal and practice characteristics. Satisfaction and compliance with the program was evaluated to assess feasibility.

**Results:**

The Training Group showed modest improvement compared to the Waitlist Group in attitudes (Δ =6.2, *p* < .001) and skills (Δ =10.0, *p* < .001) subscores, but not the use subscore (Δ = −2.3, *p* = .470). The majority of participants agreed that the educational program was ‘relevant to their profession’ (84 %) and ‘was worthwhile’ (82 %). Overall, engagement in the online program was less than optimal, with 48 % of the Training Group, and 42 % of the Waitlist Group completing all 3 of the program courses.

**Conclusions:**

Online EBP training leads to modest improvements in chiropractors’ EBP attitudes and skill, but not their use of EBP. This online program can be delivered to a wide national audience, but requires modification to enable greater individualization and peer-to-peer interaction. Our results indicate that it is feasible to deliver an online EBP education on a broad scale, but that this mode of education alone is not sufficient for making large changes in chiropractors’ use of EBP.

## Background

Evidence-based practice (EBP) has been widely adopted as a standard by all health disciplines. Defined as a systematic approach for integrating the best research evidence with clinical expertise and patients’ unique values and circumstances [1], it requires life-long self-directed learning on the part of the healthcare practitioner [2].

Interest in EBP among chiropractors, one of the most frequently-used Complementary and Integrative Health (CIH) disciplines in the US [3], has shown tremendous growth in recent years [4]. While there is some evidence to suggest that chiropractors and other integrative healthcare providers might be resistant to EBP [5, 6], a recent study by our group found that US chiropractors had generally positive attitudes toward EBP [7]. However, the same survey revealed that US chiropractors reported low self-perceived use of EBP in their clinical practices.

There are barriers to chiropractors engaging in EBP, including lack of training, skills and resources, as well as time constraints and concerns regarding the relevance of existing research [7]. These challenges are similar to those experienced by other complementary and conventional healthcare professionals [8, 9]. The rapidly growing volume of new research information further complicates the ability of practitioners to stay up-to-date with the best available research. A commonly advocated step in overcoming barriers to EBP uptake is to provide educational programs that serve 2 purposes: 1) to motivate individuals by enhancing EBP-related attitudes and beliefs, and 2) to increase capability by improving EBP-related knowledge and skills [2].

Online education programs have been shown to be as effective as in-person instruction for improving attitudes and knowledge of EBP among healthcare practitioners [2] and this mode of delivery offers a viable and sustainable solution for teaching consistent EBP content to learners over time and across multiple geographical locations [10]. Such programs also offer learners flexibility in terms of pacing and availability, advantages particularly useful for addressing health practitioners’ time constraints. The existing literature examining the effectiveness of EBP online education programs is limited however in both quantity and quality [2], especially in the CIH professions, warranting further rigorous study.

Our long term goal is to develop a comprehensive EBP literacy campaign on a national scale for chiropractors and other CIH providers. To this end, our study had 3 aims: 1) develop an online distance-learning program on the principles of evidence-based practice (EBP) for chiropractic providers; 2) test the effectiveness of the online program on the attitudes, skills, and use of EBP in a random sample of chiropractors; and 3) determine the feasibility of expanding the program for broader-scale implementation on a national basis.

The study adopted an exploratory, multi-phase, multi-method approach. We previously published the results of a national cross-sectional survey of US chiropractors, which comprised the first phase of the study and captured a baseline measure of EBP literacy [7]. The second phase of the study reported here consisted of a randomized controlled trial (RCT) comparing the effectiveness of an online education intervention with a wait-list control and assessing program feasibility. The third phase consisted of a descriptive, qualitative investigation of chiropractors’ perceived barriers and facilitators to participation; this will be reported in a forthcoming manuscript. Our hypotheses for this randomized trial were that an online EBP educational program would: 1) would be effective compared to a waitlist control in improving the attitudes, skills, and use of EBP in clinical chiropractic practice and 2) be feasible to implement.

Our study was designed to explore several questions and “unknowns” about the processes and outcomes of online EBP training. First, we found a paradoxical discordance between good attitudes but poor use of research evidence, which begged the question “why”? Secondly, although online educational programs have been shown to be generally effective, we wanted to know if an online educational program specifically focused on research and EBP would be effective in improving chiropractors’ level of EBP literacy and use. Lastly, we wanted to explore questions about which attributes of our online educational program would private practice chiropractors find useful, as well as the barriers and facilitators to completion of the online program.

## Methods

### Research design

The study was an open-label, prospective, parallel-groups randomized controlled trial with a delayed waitlist control. Volunteers came from a random sample of chiropractors who completed the first phase of the study. They were randomized using a computer-generated algorithm, prior to which group allocation was concealed from all participants and study personnel. The study was funded by the National Institutes of Health/National Center for Complementary and Integrative Health (R21 AT007547), with Institutional Review Board approval (exempt status) granted by the University of Pittsburgh (PRO12060417) and all other participating institutions. This study was conducted January 2013–September 2014.

### Participants

To participate in this RCT, individuals had to hold a doctor of chiropractic degree, reside in the US, have Internet access, have a valid email address, and have participated in the cross-sectional national survey. There were no participant exclusion criteria.

### Recruitment

Participants were recruited from an original pool of 1,314 chiropractors who completed a cross-sectional national survey [7]. We sent e-mail recruitment notices to a random sample of 700 of the 1,314 survey participants. The e-mail message included a brief explanation about the trial and an invitation to participate. A total of 293 chiropractors (42 %) responded to the email invitation and gave informed consent to be randomized.

### Interventions

The online educational program was developed and adapted from competencies and resources created through previous National Center for Complementary and Integrative Health funded EBP educational grants awarded to four chiropractic institutions [11–15]. Participants were randomly assigned to either immediate access to an online educational program (training group) or a wait-list control group. Following randomization, participants in the training group were sent an e-mail with a link to the host website to register for the program and to create a user account; this provided access to the EBP program at no cost. Participants in the waitlist group were sent a different e-mail explaining that they would be receiving instructions about registration within 9 months. Both groups were given 30 days to register, after which the registration process was closed. The program was delivered on a Moodle learner management system at the host website.

The EBP educational program was delivered online over a 7-month period for each group. The overall goal was to provide foundational skills for practitioners to become ‘informed consumers’ of research [16]. Program competencies addressed those considered foundational for enabling EBP [17], such as basic statistics, introduction to types of research, and an emphasis on ‘information mastery’ [16], as well as competencies advocated by the Sicily statement [18]. This includes 1) formulating an answerable question to a clinical problem; 2) finding the best available evidence; 3) critically appraising that evidence; 4) interpreting and applying the results; and 5) evaluating or auditing the outcome [13].

To incentivize participation, up to 10 h of continuing education credits were available to participants from qualifying states. E-mail reminders were sent 2 to 4 times per month to encourage participation, and phone calls to stimulate interest were made to the subset of participants who registered for the online program, but did not complete any courses. The EBP educational program consisted of two parts: 1) a series of online educational courses and 2) 4 monthly online booster lessons.

### Online courses

The first part of the educational intervention consisted of a series of 15 to 40-min modules, divided into 3 general courses. The entire program contained a total of 18 modules and required an estimated total of 10 h to complete. While participants were encouraged to complete the courses at their own pace over 2 months, they were provided program access for the entire 7-month intervention period. The targeted learning objectives for the program focused on the EBP topics summarized in Table 1. The online modules were developed as part of an earlier project (R25AT003582) [4, 10] using a design-based research approach [19] focused on four adult learning theories: 1) events of instruction, 2) cognitive load, 3) dual processing and 4) ARCS theory of motivation [20–24]. The modules were designed using the reusable learning object (RLO) model, where RLOs are small, self-contained units of instruction that cover a limited set of related learning objectives. Short quizzes were provided at the end of each module to foster further learning.

**Table 1:** Detailed descriptions of the individual online educational modules and booster lessons

Educational modules	Booster lessons
COURSE 1: OVERVIEW OF EVIDENCE-INFORMED PRACTICE (4 modules) • Evidence-Informed Practice (EIP) (20 mins) • Introduction to Research (25 mins) • Clinical Experience (20 mins) • Patient Presentation (15 mins)	BOOSTER LESSON 1: ASK CLINICAL QUESTIONS (30 mins) • Clinical scenario presented • Developing clinical questions • Creating a PICO** • Choosing keywords for PubMed search
COURSE 2: TYPES OF RESEARCH (7 modules) • Research Overview (30 mins) • Basic Science Research (20 mins) • Randomized Clinical Trials (35 mins) • Introduction to Observational Research (15 mins) • Types of Observational Research (30 mins) • Quantitative and Qualitative Research (40 mins) • Summary Research (40 mins)	BOOSTER LESSON 2: ACQUIRE EVIDENCE (30 mins) • Clinical scenario presented • Develop a PICO** and keyword search terms • PubMed search strategies • Video of EIP expert performing a PubMed search
COURSE 3: USING EVIDENCE IN PRACTICE (7 modules) • Research in Clinical Practice (35 mins) • Asking Clinical Questions (30 mins) • Assessing Articles about Treatment (40 mins) • Assessing Summary Research (35 mins) • Measuring Clinical Outcomes (30 mins) • Outcome Measurement Tools (20 mins) • Experts (20 mins)	BOOSTER LESSON 3: APPRAISE THE EVIDENCE (30 mins) • Results of PubMed search are presented • Open access article is found and reviewed • Worksheet is used as a guide to appraise article • Emphasis on determining relevance and validity
	BOOSTER LESSON 4: APPLY THE EVIDENCE IN PRACTICE (30 mins) • Emphasis on finding systematic reviews and meta-analyses • Review of an open-access meta-analysis • Discussion about quality and strength of evidence ratings • Use of pre-appraised evidence sources • Applying evidence in clinical practice

The bullets in the left hand column describe the content of each individual module within the 3-course series that comprise the Foundations of Evidence-Informed Practice (FEIP) program. The right column contains descriptions of the 4 monthly online booster lessons that were developed to enhance the FEIP program

*Abbreviations*: **PICO Population, Intervention(s), Comparison(s) and Outcome(s)

### Booster lessons

Four online booster lessons, consisting of 30-min interactive presentations, provided opportunities for participants to review and practice their EBP skills (Table 1). Three months after the start of the intervention, participants were sent a monthly e-mail with a link to sign in to one of four online ‘booster lessons’ hosted on the Moodle platform. Each lesson consisted of a narrated PowerPoint presentation, which presented a case, posed critical-thinking questions, and offered exercises to complete using a worksheet supplied as a PDF attachment. The design of these lessons adhered to learning and motivational theories (with particular focus on relevance and confidence), as well as applied social learning theory, which posits that individuals learn by observing and imitating others [21]. Experts from the field (peer opinion leaders) were recruited to narrate and model desired behaviors in the booster lessons.

### Data collection and measures

We collected demographic and baseline EBP information using online self-report questionnaires during our national cross-sectional survey [7]. Our effectiveness measures were the three subscales of the Evidence-Based Practice Attitude and Utilization SurvEy (EBASE), a self-report instrument to assess providers’ attitudes, skills and use of EBP [20]. The instrument has demonstrated good internal consistency, content validity, and acceptable test-retest reliability [25]. Three sections of this instrument generate subscores: Parts A (Attitudes), B (Skill) and D (Use). These subscores were used as dependent variables in statistical models to explore the effectiveness of the online education program in making changes to these outcomes. All participants were asked to complete 3 EBASE surveys over the course of the trial; baseline, 9 months (Time 2) and 16 months (Time 3).

Feasibility data collection included course and booster session completion rates. A program evaluation survey was also administered to gain insight as to participants’ satisfaction with the program. The survey included 23 items addressing the educational program overall, and the online course modules and boosters specifically, using a 5 point Likert scale (strongly disagree, disagree, neutral, agree, strongly agree).

### Statistical analysis

Power analysis showed that a sample size of n = 125 per group would yield 88 % power to detect a between-groups, standardized effect size of 0.4 on E-BASE subscore changes. We achieved this projected enrollment goal by randomizing a total of N = 293 participants; n = 147 in the Training Group and n = 146 in the Waitlist Group.

Descriptive statistics, including measures of central tendency and variability, were generated to examine the demographic characteristics and EBASE scores for the immediate access and waitlist groups. We examined the effectiveness of the educational program by comparing the differences in the between-group changes in EBASE attitudes, skills, and use subscores from baseline to Time 2 (9 months). We used general linear models with robust standard errors and controlled for baseline EBASE subscore value, gender, practice focus, education, number of patients seen daily, and years in practice. These clinically relevant covariates were each correlated with the outcomes (p < 0.05). Prior to these analyses, we rescaled each EBASE subscore to be on a 100 point scale (0 = minimum; 100 = maximum) by normalizing the data and multiplying by 100. This linear transformation was conducted to facilitate ease of interpretation of the results by using the same scale for the 3 subscores. All statistical analyses were performed using SPSS 22 (IBM Corp, Armonk, NY, USA) with statistical significance set to .05.

Although the primary endpoint for the between-group analysis was Time 2, a third EBASE (Time 3) was administered at 16 months, for two reasons. First, we wanted to explore descriptively the within-group changes in EBASE subscores following the program for the Wait-list Group (Time 3 – Time 2), to see if it was comparable the change following the program in the Training Group (Time 2 – Time 1). Second, we wanted to explore the sustainability of the within-group changes in the Training Group, 7 months after they had completed the educational intervention (Time 3 – Time 2).

Feasibility was assessed by calculating frequencies and percentages of the number of courses and booster sessions completed for each group. These descriptive feasibility data were collected at Time 2 for the Training Group, and at Time 3 for the Waitlist Group. We tracked the total number of people in each group who completed each online course and booster lesson, which was reported as the frequency. We then divided the number of completed courses and booster lessons by the total number of people in that group to arrive at the completion rates.

To ease interpretation of the program evaluation surveys, we created dichotomous variables by collapsing the response categories ‘strongly agree’ and ‘agree’ into one ‘agree’ variable, and combining ‘neutral’, ‘disagree’, and ‘strongly disagree’ responses into one ‘disagree’ variable. Frequencies and percentages were then calculated using these dichotomous variables for each group.

## Results

### Participant characteristics

Table 2 summarizes the demographic characteristics of study participants, and shows that the groups were generally balanced at baseline. Participants had a mean age of 45.6 (±11.8) years, and tended to be white (96.6 %) and male (79.7 %). On average, participants had been in practice for 16.2 (±10.3) years and consulted 20.2 (±13.5) patients daily. Over 70 % had a practice with a musculoskeletal focus. Participants were also generally balanced with respect to their mean and median baseline E-BASE subscores (Table 3), although the Waitlist Group showed slightly higher baseline values.

**Table 2:** Demographic characteristics by group

Variable	Training (*n* = 147)	Waitlist (*n* = 146)	Overall (*N* = 293)
Age in years, mean (SD)	45.7 (11.3)	45.5 (12.5)	45.6 (11.8)
Sex, no. (%)			
Female	24 (16.3)	35 (24.3)	59 (20.3)
Male	123 (83.7)	109 (75.7)	232 (79.7)
Race, no. (%)			
White	142 (96.6)	139 (96.5)	281 (96.6)
Other (Black, Asian, and/or American Indian)	5 (3.4)	5 (3.5)	10 (3.4)
Clinical experience, mean (SD)			
Years in practice	16.3 (9.8)	16.0 (10.6)	16.2 (10.3)
Number of patients seen daily	21.9 (14.9)	18.5 (11.6)	20.2 (13.5)
Education level before DC degree, no. (%)			
High School, Associate Degree/Some college	25 (17.0)	23 (16.0)	48 (16.5)
Bachelor’s Degree	107 (72.8)	87 (60.4)	194 (66.7)
Master’s Degree/Some grad work, Doctorate	15 (10.2)	34 (23.6)	49 (16.8)
Clinical Focus, no. (%)			
Musculoskeletal	98 (66.7)	110 (76.4)	208 (71.5)
Non-musculoskeletal	49 (33.3)	34 (23.6)	83 (28.5)
Geographic Location, no. (%)			
City	48 (32.7)	44 (30.6)	92 (31.6)
Suburban	72 (49.0)	77 (53.5)	149 (51.2)
Rural	27 (18.4)	23 (16.0)	50 (17.2)

*Abbreviations*: *DC* Doctor or Chiropractic, *SD* Standard Deviation

**Table 3:** Sub-scores from EBASE rescaled to a range of 0–100

		Attitudes		Skills		Use	
		Training	Waitlist	Training	Waitlist	Training	Waitlist
Baseline	Mean (SD)	70.0 (20.0)	69.1 (23.4)	49.2 (21.9)	53.6 (20.6)	41.4 (27.9)	45.7 (27.1)
	Median (IR)	66.7 (25.0)	70.8 (29.2)	47.6 (30.9)	53.6 (26.2)	29.1 (37.5)	37.5 (40.6)
Time 2	Mean (SD)	69.4 (22.0)	65.3 (20.6)	56.4 (20.0)	49.0 (21.2)	40.5 (25.9)	43.3 (28.2)
	Median (IR)	73.9 (31.5)	65.2 (21.7)	57.1 (29.2)	50.0 (26.2)	33.3 (22.9)	33.3 (40.6)
Time 3	Mean (SD)	69.7 (21.7)	73.1 (20.2)	53.2 (20.8)	55.4 (22.4)	42.0 (24.5)	46.3 (25.8)
	Median (IR)	73.9 (30.4)	78.3 (30.4)	55.2 (30.3)	52.6 (31.6)	37.5 (29.2)	41.7 (33.3)
Adjusted Group Differences (†Baseline vs. Time 2)		Attitudes		Skills		Use	
Mean (95 % CI)		6.2 (1.6, 10.7)*		10.0 (5.9, 14.2)**		−2.3 (−8.6, 4.0)	

†Training vs. Waitlist: Changes in EBASE subscores from Baseline to Time 2 after controlling for gender, focus, education, number of patients seen daily, baseline EBASE subscore and years in practice

*Abbreviations*: *EBASE* Evidence Based practice Attitude and utilization Survey, *SD* standard deviation, *IR* interquartile range

*p < .01, **p < .001

**Fig. 1:**
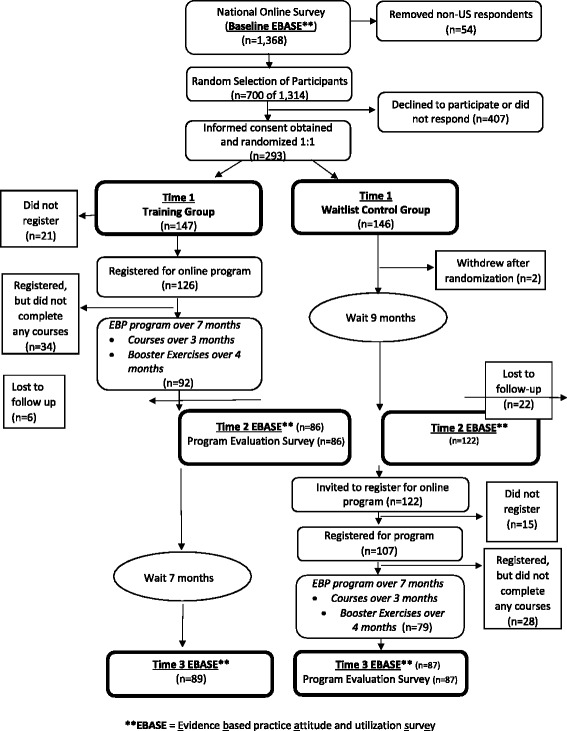
Participant flow through the DELIVER Study

Figure 1 details the flow of participants throughout the study. A total of 293 persons were randomized during the month of May 2013; 147 to the Training Group and 146 to the Waitlist Group. Two individuals immediately withdrew from the Waitlist Group after randomization, and an additional 22 were lost to follow up at Time 2, prior to the invitation to register. A number of participants in both groups did not register for the program after receiving the registration invitation (Training: n = 21, Waitlist: n = 15); this resulted in registration rates for the online education program of 86 % for those randomized to the Training Group and 73 % for those randomized to the Waitlist Group.

In terms of data collection follow up, at Time 2 (primary endpoint), 59 % of the training group completed the second EBASE and the Program Evaluation Survey, compared to 84 % of the Waitlist Group. At Time 3, 61 % of the Training Group and 60 % of the Waitlist Group completed the third EBASE questionnaire.

#### Preliminary effectiveness outcomes: EBP attitude, skills and use

Table 3 presents the three EBASE subscores for each group and at each time point. Means with standard deviations and medians with interquartile ranges are reported, as well as between-group comparisons with 95 % confidence intervals. Medians were reported in addition to means, due to the highly skewed distributions of the Attitudes and Use subscores. Attitude subscores favored the Training Group with an adjusted mean difference of 6.2 (p = .008) for the multi-covariate model. Skills subscores also demonstrated the greatest performance advantage for the Training Group (Δ =10.0, *p* < .001). There was no statistically significant difference between groups for the Use subscores (Δ = −2.3, *p* = .470). Table 3 also shows modest within-group improvements in Attitudes and Skills EBASE subscores (but not the Use subscore) for the Waitlist Group from Time 2 to Time 3, comparable to those between Baseline and Time 2 in the Training Group.

#### Feasibility outcomes

Feasibility was measured by rates of online EBP course completion and the monthly booster session completion rates. Generally the course and booster session completion rates among those randomized was low, with only 71 (48 %) randomized to the Training Group completing all three courses compared to 61 (42 %) in the Waitlist Group. For the boosters, 79 (54 %) of participants randomized to the Training group compared to 59 (41 %) in the waitlist, failed to participate in any booster lessons. Only 23 (16 %) in the Training Group and 35 (24 %) in the Waitlist Group completed all four booster lessons.

Table 4 illustrates the responses from participants about the program evaluation survey. The majority of survey respondents agreed that the *overall educational program* was ‘relevant to their profession’ (84 %) and ‘was worthwhile’ (82 %). Participant views regarding specific aspects of the online course modules and the booster lessons are also illustrated in Table 4. In general, the comments were more favorable regarding the educational modules compared to the booster lessons.

**Table 4:** Responses to program evaluation survey of the EBP online educational program

*N* = 171	Agree	
Evaluation survey response	*n*	%
The Overall Program…		
Was relevant to profession	144	84.2
Held my interest	123	71.9
Was worthwhile	140	81.9
^a^The Modules…		
Were easy to use	131	76.6
Graphic/captions helped me learn	135	78.9
Text helped me learn	140	81.9
Practice and feedback helped me learn	139	81.3
Examples helped me learn	139	81.3
Quizzes helped me learn	130	76.0
Length is just right	106	62.0
Design is appealing	118	69.0
Amount of text is easy to get through	125	73.1
Interaction is interesting	132	77.2
The Boosters…		
Were easy to use	108	63.2
Were easy to understand	111	64.9
Had right amount of information	102	59.6
Took the appropriate amount of time	98	57.3
Narration helped me learn	112	65.5
Were relevant to profession	108	63.2
Held my interest	101	59.1
Worksheet helped me learn	97	56.7
Reinforced skills	110	64.3
Were worthwhile	109	63.7

^a^Modules were components of the online courses (see Table 1)

## Discussion

This is one of the first studies to systematically explore the effectiveness and feasibility of delivering an online EBP educational program for chiropractors, one of the most widely utilized CIH disciplines in the United States [26].

### Preliminary effectiveness

Significant differences between the training and waitlist groups were observed in self-reported EBP attitudes and skills, but there was no meaningful change in EBP use following exposure to the educational program. However, the magnitude of these significant group differences were only modest, and given that no established standard exists for what constitutes a meaningful difference in EBP outcomes, the potential impact of these findings is unknown.

The modest improvement in EBP attitudes and skills, and the absence of any change in EBP use were unexpected findings. This is in light of literature showing improvements in EBP knowledge, skills, attitudes, and behaviors following EBP training in other health disciplines, including allied health [27], nursing [28] and medicine [29]. There are several possible explanations for why our results differed from other studies.

First, it is possible that the small change in attitudes was due to self-selection bias and an associated ceiling effect. That is, participants were already favorably inclined towards EBP and motivated to take part in the study, resulting in high pre-intervention attitude sub-scores with little room for improvement. These same factors might also explain the modest change in EBP skills. Another explanation for the observed outcomes is that the previously developed online courses were designed as a ‘companion’ intervention to be used with other more interactive educational venues such as classes, workshops, and other formats [30]. While the booster lessons were intended to complement the courses, they were designed to be done asynchronously and independent of an instructor. The intent was to make these lessons more amenable to broad scale implementation. This limited the ability to provide the individualized feedback, collaborative learning, or instructor-student interaction. These are considered important educational strategies from an adult learning perspective [17, 20, 31, 32].

Further, if our EBP educational program is considered within the context of relevant behavioral theories, the lack of social support in the program may be a critical and absent component. While the program did provide opportunity to enhance EBP capabilities critical for enacting and enabling EBP behaviors [33, 34], the format failed to provide in-depth social support to participants, which in the form of interaction with peers, peer-coaches and/or opinion leaders may be important for advancing EBP in health professionals [35].

Our choice of outcome measure may also have influenced the observed results. Ideally, outcome measures should be both psychometrically sound and mapped specifically to what an intervention aims to achieve [36]. While the EBASE had been previously validated for CAM professions, there were some EBASE questions that did not map well to a number of our EBP learning objectives. For example, the EBASE items that were related to conducting clinical research and systematic reviews, contrasted with our program’s focus on information mastery. Conversely, there were learning objectives addressed in our online modules and booster sessions that were not measured in the EBASE. Examples include the sections of our modules that covered the types of research questions answered by different research designs and statistical concepts.

Given the educational program was considered foundational in nature, we did not anticipate large changes in the EBASE ‘use’ domain. In fact, it is unlikely that >any educational program will result in important changes in research use due to the inherent complexity of EBP-related behaviors [2, 8]. Instead, EBP educational programs should be viewed as comprising only one necessary component of behavioral change: the opportunity to address practitioners’ capabilities, specifically attitudes, knowledge and skills. Multi-factorial strategies will be required to address other individual and system-related issues in an ongoing and sustainable manner [2, 36].

### Feasibility

Regarding our feasibility aims, we were able to successfully develop and host a series of online educational modules and deliver monthly booster lessons to almost 300 chiropractors in different geographic regions of the US. However, we found generally poor compliance with completion of the online courses and booster lessons. The engagement data illustrated that a number of individuals (14–26 %) failed to register for the educational program after randomization. In this study, participants were required to take a number of online steps (clicks) in order to proceed from registration to commencement of the educational program, the purpose of which was to accommodate the processing of continuing education credits. This may have proved un-motivating to some, especially those unaccustomed to online education formats [37].

The engagement rates for those that *did* register for the program was also somewhat disappointing, especially given efforts encouraging participation via e-mail and telephone reminders. Of those that registered for the program, 55–63 % completed at least one course with 42–48 % completing all three courses. Participation in the booster lessons was far less, with 32–33 % completing at least one lesson, and only 16–24 % completing all four. Indeed, motivating individuals to complete online courses is a well-recognized challenge for education. Others have noted a 10–20 % greater drop-out rate for online courses compared to traditional classroom environments [38], and completion rates of massive open online courses (MOOCs) is a dismal 2–14 % [39].

Noteworthy is that of those in both groups who completed one course in our program (n = 171), the majority went on to complete all three (n = 132). This observation, particularly in light of other online participation rates, suggests our program was successful once individuals overcame the initial obstacles to commencement.

### Strengths and limitations

A strength of this study is the randomized, waitlist-controlled design with careful attention paid to the feasibility of implementation. Further, given that there has been little research investigating the effectiveness of online learning for improving EBP attitudes, knowledge, and behaviors [2], our study makes an important contribution to the EBP education literature by providing preliminary information regarding effectiveness. Additionally, we have provided a description of the intervention [10] in accordance with the Guideline for Reporting Evidence-based practice Educational Intervention and Teaching (GREET), the purpose of which is to guide the design and interpretation of EBP educational interventions [2, 28, 40, 41]. This will aid others in interpreting the results, and optimizing and testing future EBP education programs. Limitations of the study include the generally low data collection follow up and intervention engagement rates. This study was designed to be pragmatic in nature, reflecting how an EBP online education program could be implemented in real-world settings. Future studies aiming to establish the efficacy and effectiveness of such programs should take additional efforts to bolster participation.

### Implications

The results of this study have several implications for those embarking on future EBP educational initiatives, particularly those aimed at broader scale implementation regardless of healthcare discipline. Future programs should consider including the use of moderated online discussions and other interactive methods as ways to provide individualized feedback, collaborative learning and instructor-student interaction; all of which are known to facilitate adult learning [17, 20, 42, 31, 32]. Further, such methods targeting interaction with peers, peer-coaches and/or opinion leaders should be considered [29]. Importantly, those aiming to rigorously evaluate the effectiveness of EBP programs should carefully weigh the strengths and limitations of the range of existing EBP outcome measures, in order to ensure alignment with their program competencies [17, 18]. Also, greater attention should be paid to stream-lining registration processes, especially with online programs to facilitate ease of use. Finally, to optimize participation in future EBP programs, a better understanding of what motivates practitioners to engage in online educational activities would be advantageous. A manuscript addressing barriers and facilitators to participation in this study will be reported separately.

## Conclusion

This exploratory study found that an EBP educational program can be delivered in an online format; however relatively poor engagement suggests several barriers exist which need to be considered in future research and implementation efforts. Our online educational program resulted in small positive improvements in chiropractors’ attitudes and skills in EBP, but not their level of EBP uptake (use). This suggests that such online programs can provide an opportunity to enhance practitioners’ motivation and capacity for EBP, but that online education alone is not sufficient to make large changes in EBP use behaviors. The feasibility and effectiveness results from this study can be used to design and refine future, and more robust EBP training and implementation initiatives that can have larger impact on a broader scale.

The online courses described in this manuscript are freely available at http://www.csh.umn.edu/research/foundations-evidence-informed-practice-modules.

## References

[1] Sackett DL, Straus SE, Richardson WS, Rosenberg W, Haynes RB (2000). Evidence-based medicine.

[2] Young T, Rohwer A, Volmink J (2014). What are the effects of teaching evidence-based health care (EBHC)? Overview of systematic reviews. PLoS One.

[3] Clarke TC, Black LI, Stussman BJ (2015). Trends in the use of complementary health approaches among adults: United States, 2002–2012. Natl Health Stat Rep.

[4] Evans R, Maiers M, Delagran L (2012). Evidence informed practice as the catalyst for culture change in CAM. Explore (NY).

[5] Hall G (2011). Attitudes of chiropractors to evidence-based practice and how this compares to other healthcare professionals: A qualitative study. Clin Chiropr.

[6] Suter E, Vanderheyden LC, Trojan LS, Verhoef MJ, Armitage GD (2007). How important is research-based practice to chiropractors and massage therapists?. J Manipulative Physiol Ther.

[7] Schneider MJ, Evans R, Haas M (2015). US chiropractors’ attitudes, skills and use of evidence-based practice: A cross-sectional national survey. Chiropr Manual Ther.

[8] Ubbink DT, Guyatt GH, Vermeulen H. Framework of policy recommendations for implementation of evidence-based practice: a systematic scoping review. BMJ open. 2013;3(1). doi:10.1136/bmjopen-2012-001881. 10.1136/bmjopen-2012-001881 PMC3563143 23355664

[9] Sadeghi-Bazargani H, Tabrizi JS, Azami-Aghdash S (2014). Barriers to evidence-based medicine: a systematic review. J Eval Clin Pract.

[10] Delagran L, Vihstadt C, Evans R (2015). Aligning Theory and Design: The Development of an Online Learning Intervention to Teach Evidence-based Practice for Maximal Reach. Glob Advances Health Med.

[11] Zwickey H, Schiffke H, Fleishman S, Haas M, Cruser DA, Lefebvre R, Sullivan B, Taylor B, Gaster B. Teaching evidence-based medicine at complementary and alternative medicine institutions: strategies, competencies, and evaluation. J Altern Complement Med. 2014;20(12):925–31. 10.1089/acm.2014.0087 PMC4270143 25380144

[12] Sullivan BM, Furner SE, Cramer GD (2014). Development of a student-mentored research program between a complementary and alternative medicine university and a traditional, research-intensive university. Acad Med.

[13] Haas M, Leo M, Peterson D, Lefebvre R, Vavrek D (2012). Evaluation of the effects of an evidence-based practice curriculum on knowledge, attitudes, and self-assessed skills and behaviors in chiropractic students. J Manipulative Physiol Ther.

[14] Lefebvre RP, Peterson DH, Haas M, Gillette RG, Novak CW, Tapper J, Muench JP. Training the evidence-based practitioner: university of Western States document on standards and competencies. J Chiropr Educ. 2011;25(1):30–7. 10.7899/1042-5055-25.1.30 PMC3113621 21677870

[15] Long CR, Ackerman DL, Hammerschlag R, Delagran L, Peterson DH, Berlin M, Evans RL. Faculty development initiatives to advance research literacy and evidence-based practice at CAM academic institutions. J Altern Complement Med. 2014;20(7):563–70. 10.1089/acm.2013.0385 PMC4086219 24936915

[16] Slawson DC, Shaughnessy A (2005). Teaching evidence-based medicine: Should we be teaching information management instead?. Acad Med.

[17] Mizerow J, Mezirow and Associates (2000). Learning to Think Like an Adult: Core Concepts of Transformation Theory. Learning as Transformation.

[18] Dawes M, Summerskill W, Glasziou P (2005). Sicily statement on evidence-based practice. BMC Med Educ.

[19] Dolmans DH, Tigelaar D (2012). Building bridges between theory and practice in medical education using a design-based research approach: AMEE Guide No. 60. Med Teach.

[20] Gagne RM (1977). The conditions of learning.

[21] Sweller J (2011). The psychology of learning and motivation: cognition in education (55th Edition).

[22] Keller JM (2009). Motivational Design for Learning and Performance: the ARCS Model.

[23] Keller JM, Suzuki K (2004). Learner motivation and E-learning design: a multi-nationally validated process. J Educ Media.

[24] Bandura A (1997). Self-efficacy: The exercise of control.

[25] Leach MJ, Gillham D (2008). Evaluation of the evidence-based practice attitude and utilization survey for complementary and alternative medicine practitioners. J Eval Clin Pract.

[26] Weeks WB, Goertz C, Meeker W, Marchiori D (2015). Public Perceptions of Doctors of Chiropractic: Results of a National Survey and Examination of Variation According to Respondents’ Likelihood to Use Chiropractic, Experience With Chiropractic, and Chiropractic Supply in Local Health Care Markets. J Manipulative Physiol Ther.

[27] Dizon JMR, Grimmer-Somers KA, Kumar S (2012). Current evidence on evidence-based practice training in allied health: a systematic review of the literature. IntJ Evid Based Healthc.

[28] Mooney S (2012). The effect of education on evidence-based practice and nurses’ beliefs/attitudes toward and intent to use evidence-based practice. Doctoral Dissertation.

[29] Sprague S, Pozdniakova P, Kaempffer E, Saccone M, Schemitsch EH, Bhandari M (2012). Principles and Practice of Clinical Research course for surgeons: an evaluation of knowledge transfer and perceptions. Can J Surg.

[30] Evans R, Delagran L, Maiers M (2011). Advancing evidence informed practice through faculty development: the Northwestern Health Sciences University model. Explore (NY).

[31] Akyol Z, Garrison DR (2013). Educational Communities of Inquiry: Theoretical Framework, Research and Practice.

[32] Johnson DW, Johnson RT, Smith KA (1998). Cooperative learning returns to college what evidence is there that it works?. Change: the magazine of higher learning.

[33] Michie S, van Stralen MM, West R (2011). The behaviour change wheel: a new method for characterising and designing behaviour change interventions. Implement Sci.

[34] Cialdini RB (2007). Influence: the Psychology of Persuasion.

[35] Frantsve-Hawley J, Meyer DM (2008). The evidence-based dentistry champions: a grassroots approach to the implementation of EBD. J Evid Based Dent Pract.

[36] Craig P, Dieppe P, Macintyre S (2013). Developing and evaluating complex interventions: the new Medical Research Council guidance. Int J Nurs Stud.

[37] Greenhalgh T (2001). Computer assisted learning in undergraduate medical education. BMJ.

[38] Herbert M. Staying the Course: A Study in Online Student Satisfaction and Retention. Online Journal of Distance Learning Administration*.* 2006; (9)4

[39] Perna L, Ruby A, Boruch R, et al. The Life Cycle of a Million MOOC Users. Presentation December 5, 2013: MOOC Research Initiative Conference. Powerpoint file, accessed 17 Jan 2016 at: http://k12accountability.org/resources/Online-Education/perna_ruby_boruch_moocs_dec2013.pdf

[40] Phillips AC, Lewis LK, McEvoy MP (2014). A Delphi survey to determine how educational interventions for evidence-based practice should be reported: stage 2 of the development of a reporting guideline. BMC Med Educ.

[41] Phillips AC, Lewis LK, McEvoy MP (2014). A systematic review of how studies describe educational interventions for evidence-based practice: stage 1 of the development of a reporting guideline. BMC Med Educ.

[42] Newman I, Benz CR. Qualitative-quantitative Research Methodology: Exploring the Interactive Continuum. Carbondale, IL: Southern Illinois University Press; 1998. p.158-165.

